# Decoding SR protein regulation: kinases, phosphatases, and therapeutic targeting strategies

**DOI:** 10.1007/s13402-026-01168-8

**Published:** 2026-02-04

**Authors:** Nasi Liu, Fleur van der Ende, Bob van de Water, Sylvia E. Le Dévédec

**Affiliations:** Division of Cell Systems and Drug Safety, Leiden Academic Centre for Drug Research (LACDR), Leiden University, Einsteinweg 55, Leiden, 2333 CC The Netherlands

**Keywords:** RNA splicing, SR proteins, Cancer, Phosphorylation

## Abstract

RNA splicing is a fundamental cellular process that transforms precursor messenger RNA (pre-mRNA) into mature messenger RNA (mRNA) by removing non-coding introns and rejoining coding exons. Serine/arginine-rich (SR) proteins, a family of RNA-binding proteins, play crucial roles in RNA splicing by recruiting essential components for spliceosome assembly. The activity of SR proteins is tightly regulated by post-translational modifications, including phosphorylation, acetylation, methylation, and ubiquitination. Among these, the dynamic balance between phosphorylation and dephosphorylation is particularly critical for modulating SR protein function. Given their involvement in cancer, SR proteins represent promising targets for therapeutic intervention. In this review, we provide a comprehensive overview of the current understanding of the regulatory networks involving kinases and phosphatases governing SR protein phosphorylation. We also discuss the existing therapeutic strategies using small-molecule inhibitors aimed at regulating SR protein phosphorylation in the context of cancer. In conclusion, this review highlights the importance of phosphorylation regulation in SR protein function and the RNA splicing process. Targeting SR protein phosphorylation may open new therapeutic avenues or enhance the efficacy of cancer treatments when used in combination with other drugs.

## Introduction

RNA splicing is a fundamental cellular process that plays a crucial role in the conversion of precursor messenger RNA (pre-mRNA) into mature messenger RNA (mRNA) molecules [1]. This process takes place in the nucleus and is achieved by removing the non-coding introns and splicing back together the coding exons of the pre-mRNA [1]. There are two types of splicing: constitutive splicing and alternative splicing. In constitutive splicing, the exons of the pre-mRNA transcript are ligated in the same order as they appear in the gene, whereas in alternative splicing not only the ligation order, but also the inclusion of introns/exons and splice sites can extensively vary [2]. According to RNA sequencing data, approximately 90–95% of human genes undergo alternative splicing, frequently in a manner specific to tissue type, condition, or species [3, 4]. This phenomenon ultimately leads to the translation of different splicing transcript variants and thereby contributes to the diversity and complexity of proteins produced from a single gene [5, 6].

**Fig. 1:**
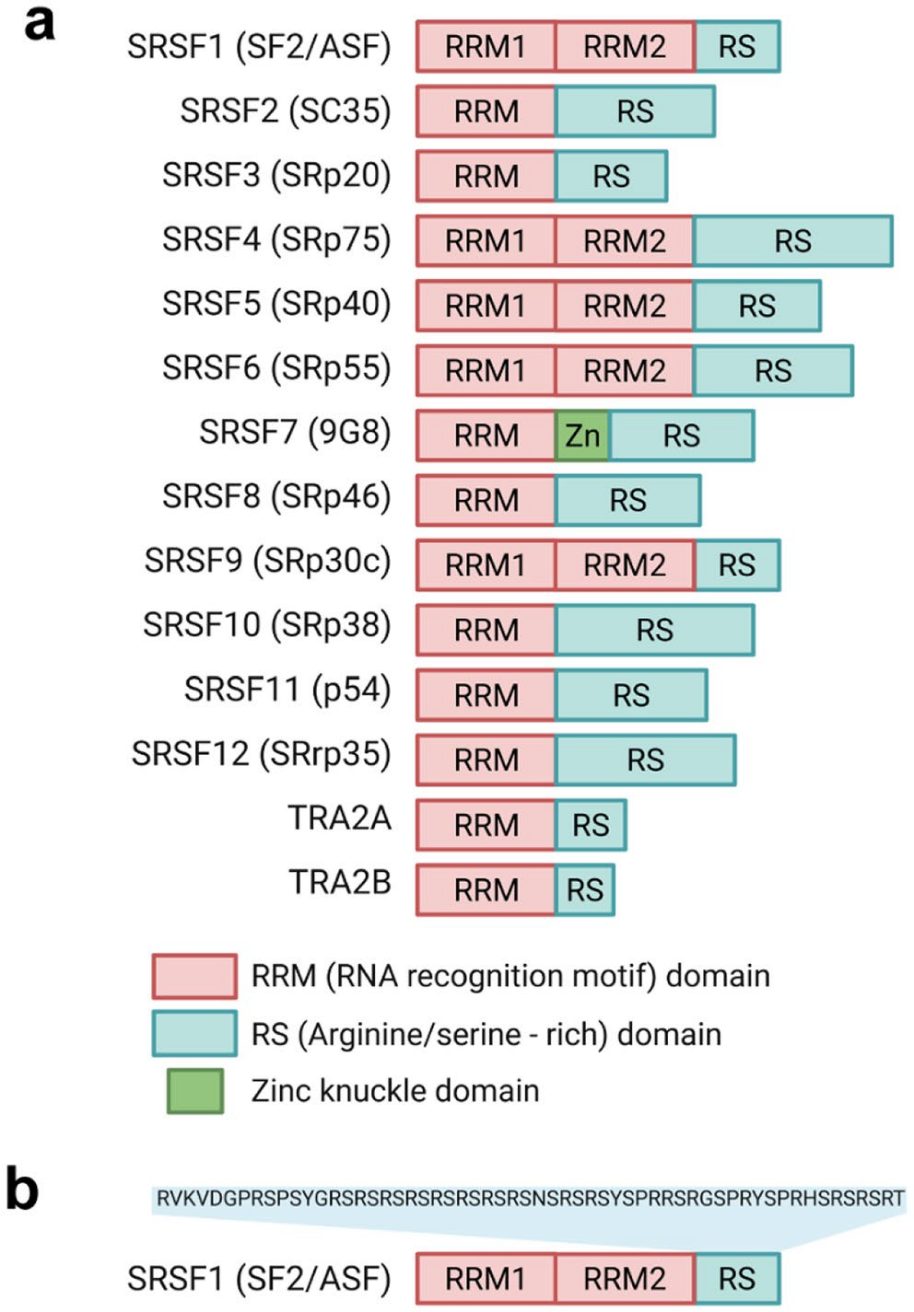
**a** Schematic representation of the major domains of the 12 canonical SR proteins (SRSF1–SRSF12) and two SR-related proteins (TRA2A and TRA2B). Each protein contains one or two RNA recognition motif (RRM) domains (red), responsible for RNA binding, and an arginine/serine-rich (RS) domain (blue), which mediates protein-protein interactions. SRSF7 uniquely contains a zinc knuckle domain (Zn, green) inserted between its RRM and RS domains. Protein names are followed by common aliases in parentheses. **b** Detailed view of the SRSF1 (SF2/ASF) domain structure showing the amino acid sequence of its RS domain. This figure was created with BioRender. com, accessed on WK28OBH1QR, 25 August 2025

Serine- and arginine-rich (SR) proteins are regulatory RNA-binding proteins (RBPs) that play essential roles in multiple gene expression pathways, primarily through their function as splicing factors involved in both constitutive and alternative splicing [7, 8]. Besides their function in RNA splicing, SR proteins also impact genomic stability, mRNA export, mRNA stability and translation [9–11]. SR proteins contain at least one N-terminal RNA recognition motif (RRM) and one C-terminal arginine/serine (RS)-rich domain spanning at least 50 amino acids, with the serine and arginine content exceeding 40% (Fig. 1) [11, 12]. The RRM domain is responsible for specific binding to the downstream target pre-mRNA, while the RS domain plays a key role in the formation and assembly of the spliceosome [13]. There are twelve classical serine-arginine splicing factors (SRSFs) within the SR protein family [12]. Some of these SRSFs are well-known for their regulatory function in RNA processing and gene expression, while others are still under-investigated [14–17]. Notably, members of the transformer (TRA) family, TRA2A and TRA2B, each containing one RRM and one RS domain, have also been classified as SR proteins and act as sequence-specific splicing activators (Fig. 1)[18, 19]. Furthermore, there are many more proteins that also carry the RS domain, known as SR-related proteins, some of which are also involved in RNA splicing [12].

The post-transcriptional and post-translational regulation that controls the expression and activity of SR protein has emerged as a critical determinant in many cellular processes [20, 21]. Post-translational modifications play a substantial role in the regulation of SR proteins. These modifications include phosphorylation, methylation, acetylation, and ubiquitination, with phosphorylation standing out as a principal contributor [22]. The SR protein kinases (SRPKs) and Cdc2-like kinases (CLKs) have been shown to effectively phosphorylate SR proteins or SR-related proteins, whereas phosphatases, such as protein phosphatase 1 (PP1), are responsible for their dephosphorylation [23–25]. In addition to SRPKs and CLKs, several cellular signaling pathways, such as the PI3K/AKT pathway, have also been implicated in directly regulating SR protein phosphorylation [26]. Furthermore, the PI3K/AKT pathway may modulate the activity of SRPKs and CLKs, thereby also indirectly influencing SR protein phosphorylation [26]. The balance between phosphorylation and dephosphorylation is essential to ensure the proper functioning of SR proteins in RNA processing. For example, phosphorylation of SR proteins is imperative for their nuclear import and subsequent regulatory roles within the spliceosome [27]. Conversely, partial dephosphorylation is necessary to facilitate the proper spliceosome assembly and function [28]. Dysregulation of phosphorylation, manifested as hyper- and hypo- phosphorylation, has been implicated in cancer development and progression [29].

In this review, we provide a comprehensive overview of the kinases and phosphatases that regulate SR protein phosphorylation, along with their intricate regulatory networks. Furthermore, we discuss the potential of targeting SR protein phosphorylation as a promising strategy for cancer therapeutics.

## Regulation of SR protein phosphorylation by kinases

Post-translational modifications (PTMs) play a crucial role in the regulation of various cellular processes by adding complexity to protein function [30]. SR proteins undergo diverse PTMs, e.g., phosphorylation, methylation, ubiquitination, and acetylation [31–34]. These modifications can alter their activity and subcellular localization, thereby influence their function in RNA processing.

**Fig. 2:**
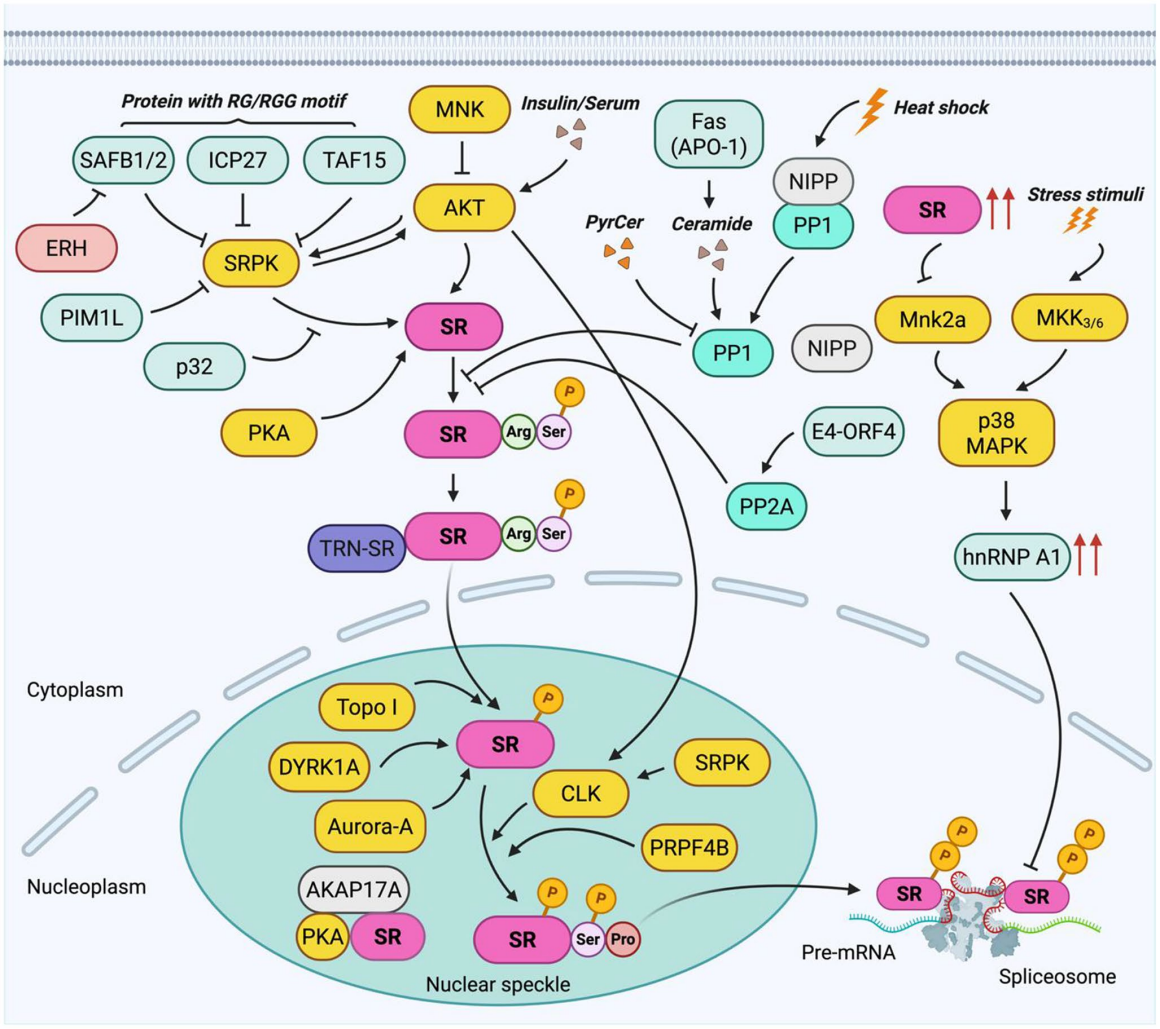
Regulatory network of the (de)phosphorylation of SR proteins. This diagram illustrates how serine/arginine-rich (SR) proteins are regulated by phosphorylation and dephosphorylation by kinases, phosphatases, cellular signals, and other factors. SR proteins are depicted in pink. Kinases and phosphatases are shown in yellow and blue green, respectively. Proteins that positively regulate SR protein function or phosphorylation are indicated in red, while those with negative regulatory effects are shown in light green. Arrow lines represent a positive relation. Stop lines represent a negative relation. In the cytoplasm, SR protein kinase (SRPK) phosphorylates serine (Ser) and arginine (Arg) residues of SR proteins, facilitating their nuclear import via transportin-SR (TRN-SR). Long isoform of PIM1 (PIM1L) and proteins containing arginine-glycine or arginine-glycine-glycine (RG/RGG) motifs, including scaffold attachment factors B1/2 (SAFB 1/2), TATA-box binding protein-associated factor 15 (TAF15) and the viral protein ICP27, negatively regulate SRPK activity. The splicing factor-associated protein p32 binds unmodified SR protein and partially inhibits SRPK1-mediated phosphorylation. Enhancer of rudimentary homolog (ERH) binds SAFB1/2, abolishing their inhibition of SRPK1 activity. Inside the nucleus, primarily within nuclear speckles, phosphorylation of SR proteins is further modulated by CDC-like kinases (CLKs) at both Ser-Arg and Ser- proline (Pro) residues. Dual-specificity tyrosine-phosphorylation regulated kinase 1A (DYRK1A), pre-mRNA processing factors kinase 4B (PRPF4B), Aurora kinase A (Aurora A), and topoisomerase I (Topo I) also contribute to SR protein phosphorylation in the nucleus. CLK-SRPK interaction further enhances Ser-Pro phosphorylation of SR proteins, promoting their release from nuclear speckles and spliceosome assembly. The phosphorylation status of SR proteins is counterbalanced by protein phosphatases 1 and 2A (PP1 and PP2A), whose activities are regulated by nuclear inhibitor of PP1 (NIPP) and the viral protein E4–ORF4. The Fas cell-surface death receptor (also known as APO-1) triggers endogenous ceramide production, which activates PP1 and results in SR protein dephosphorylation, whereas the synthetic analog C6 pyridinium ceramide (PyrCer) inhibits PP1, blocking dephosphorylation of SR proteins. AKT directly phosphorylates SR proteins upon PI3K pathway activation by insulin/serum. It also indirectly enhances SR protein phosphorylation by activating SRPK and CLK, while SRPK overexpression in turn promotes AKT signaling, establishing a feedback loop. MAPK-interacting kinase (MNK) indirectly modulate SRPK activity by negatively regulating mTORC2–AKT signaling. Protein kinase A (PKA), activated by cAMP and anchored by A-kinase anchoring protein (AKAP) 17A, colocalizes with SR proteins in nuclear speckles. It also directly interacts with SR protein and modulates its phosphorylation. Stress-activated pathways involving MAP kinase kinases 3 and 6 (MKK3/6) and p38 mitogen-activated protein kinase (p38 MAPK) regulates hnRNP A1, a functional antagonist of SR protein, altering the nuclear hnRNP A1/SRSF1 ratio and affecting alternative splicing, while SR protein modulates the MNK/p38 MAPK axis. This figure was created with BioRender. com, accessed on RZ296RZ7G8, 1 January 2026

Phosphorylation is the most extensively studied PTM of SR proteins, tightly regulated by a balance between kinases and phosphatases [28, 35]. Phosphorylation of the RS domain is crucial for the nuclear import/export, mRNA transport and spliceosome assembly, while dephosphorylation is essential for splicing catalysis, highlighting the importance of tightly controlled phosphorylation for proper RNA processing [13, 34, 35]. There are two main protein kinase families that mediate SR protein phosphorylation: SR protein-specific kinases (SRPKs) and Cdc2-like kinases (CLKs) (Table 1) [24]. Other kinases also contribute to SR protein phosphorylation, including the pre-mRNA processing factors (PRPF) family, dual-specificity tyrosine-phosphorylation regulated kinase (DYRK) family, PI3K/AKT pathway kinases, MAPK pathway kinases, cAMP/PKA pathway kinases, DNA topoisomerase I, Aurora kinase A, and snRNP-associated kinases (Table 1). Figure 2 illustrates the overview of the regulatory (de)phosphorylation network of SR proteins.

**Table 1:** Kinases regulating SR protein phosphorylation and functions

Kinases	Effects on SR protein function	Phosphorylation features	Direct/indirect regulation	Well-studied SR protein targets
SRPK family	Initiates SR protein phosphorylation to promote nuclear import; enhances interaction with U1-snRNP; primes SR proteins for spliceosome assembly	Preferential phosphorylation of Arg-Ser (RS) dipeptides	Direct	SRSF1 (major), SRSF2, SRSF3, SRSF4, SRSF5, SRSF6, SRSF7
CLK family	Phosphorylates nuclear SR proteins and releases them from speckles; increases nucleoplasmic availability of SR proteins; facilitates spliceosome assembly and dynamic splicing regulation	Phosphorylates both Arg–Ser and Ser–Pro dipeptides; RS-rich N-terminal substrate binding	Direct	SRSF1, SRSF2, SRSF4, SRSF5, SRSF6, SRSF9
PRPF family	Maintains SR protein phosphorylation within spliceosome complexes; stabilizes spliceosome B complex; supports sustained SR protein function during splicing	RS-domain phosphorylation (e.g., SRSF1)	Direct	SRSF1
DYRK family	Modulates SR protein phosphorylation and subnuclear distribution; alters SR protein localization; regulates exon inclusion/skipping decisions	Multi-site serine phosphorylation within RS domains	Direct	SRSF1, SRSF2, SRSF7
PI3K/AKT pathway kinases	Phosphorylates SR proteins and activates SRPKs/CLKs; couples growth factor signaling to splicing; enhances SR protein activity under signaling cues	RS-domain phosphorylation; activation of SRPK1 and CLK1	Both direct and indirect	SRSF1, SRSF2, SRSF4, SRSF5, SRSF7
MAPK pathway kinases	Indirect modulation via antagonistic splicing factors and AKT signaling; shifts balance between SR proteins and hnRNPs, altering splicing outcomes	Limited evidence for direct SR phosphorylation	Indirect	None clearly defined (SR proteins affected indirectly)
cAMP/PKA pathway kinases	Modulates SR protein phosphorylation and RNA binding; alters RNA binding affinity and exon selection	Phosphorylation of SR proteins (e.g., SRSF1, SRSF2)	Direct	SRSF1, SRSF2
DNA topoisomerase I (Topo I)	Phosphorylates SR proteins during transcription; enhances ESE-dependent splicing; coordinates transcription-splicing coupling	ATP-dependent RS-domain phosphorylation	Direct	SRSF6, multiple SR/SR-related proteins
Aurora kinase A (AURKA)	Direct phosphorylation of select SR proteins; modulates SR protein abundance and splicing activity during cell cycle	Phosphorylates SRSF1, SRSF2, SRSF3, SRSF	Direct	SRSF1, SRSF2, SRSF3, SRSF7
snRNP-associated kinase	Phosphorylates RS domains of snRNP and SR proteins; Supports snRNP maturation and spliceosome function	RS-domain phosphorylation of U1-70K and SRSF1	Direct	SRSF1

### SRPK family kinases

SRPK family kinases consist of three members: SRPK1, SRPK2 and SRPK3. They are localized in both the nucleus and cytoplasm [35]. The two well-studied human homologs, SRPK1 and SRPK2, share similar enzymatic activity and substrate specificity [36]. Both kinases facilitate protein–protein interaction (PPI) through SR proteins (Fig. 2) [35, 37]. SRPK3, also known as STK23, is predominantly expressed in striated muscle and is essential for muscle growth and homeostasis [38]. SRPKs overexpression induces the SR proteins redistribution from nuclear speckles to the nucleoplasm, implicating a role in spliceosome assembly regulation [24].

Despite their similarities, SRPK1 and SRPK2 exhibit distinct tissue-specific expression patterns. While they are comparably expressed in the heart, liver, lung, and kidney, SRPK2 shows significantly higher expression in the brain, whereas SRPK1 is predominantly elevated in the pancreas [39–41]. In the context of cancer, SRPK1 is overexpressed in colorectal and prostate cancer and serves as an early-stage biomarker [42, 43]. Its knockdown suppresses tumor cell growth, migration, and angiogenesis [43]. In breast cancer, SRPK1 upregulation is correlated with poor prognosis, whereas its depletion reduces metastasis to organs such as the lung and brain [44]. SRPK2 is frequently elevated in non-small cell lung cancer, colorectal cancer, and melanoma, associated with poor overall survival and prognosis. Its knockdown impairs proliferation, metastasis, and invasiveness [45–47].

#### SRPK-mediated phosphorylation is essential for the nuclear import of SR proteins

As previously discussed, SRPKs are critical regulators of SR protein function. SRPK1 and SRPK2 phosphorylate the RS domain of SRSF1, enhancing its interaction with U1-70K, a component of the U1 snRNP [36]. This facilitates the ternary complex formation essential for U1 snRNP recruitment to the 5′ splice site. Reduced SRPK1 expression or treatment with SRPKs inhibitors (e.g., SRPIN340, SPHINX31) decreases SRSF1 phosphorylation, leading to its cytoplasmic accumulation and widespread splicing defects [48, 49]. Similarly, SRPK2 knockout results in SRSF1 accumulation in the cytoplasm, supporting the role of SRPK-mediated phosphorylation in SRSF1 nuclear import [50].

Previous research has shown that the nuclear import of SR proteins is mediated by transportin-SR1 (TRN-SR1) and transportin-SR2 (TRN-SR2). The RS domain of SR proteins functions as a nuclear localization signal (NLS) [51, 52]. SRPK1-mediated RS domain phosphorylation enhances SR proteins binding to TRN-SR2 and is imperative for the TRN-SR2-driven nuclear import (Fig. 2) [53]. However, hyper-phosphorylation of the RS domain induces the redistribution of SR proteins by counteracting their retention in the nuclear speckles, leading to either a more diffuse dispersion within the nucleoplasm or accumulation in the cytoplasm [54]. TRN-SR1 contains an additional unique region in its central domain, which is not present in TRN-SR2. The latter selectively imports phosphorylated SR protein, whereas the unique domain of TRN-SR1 might influence its binding specificity with other proteins [53, 55].

#### SRPKs inhibitors

Several proteins have been reported to inhibit the phosphorylation of SRPKs’ downstream substrates and thereby characterized as SRPKs inhibitors. Proteins containing the arginine-glycine or arginine-glycine-glycine (RG/RGG) motifs, such as scaffold attachment factors B1/2 (SAFB 1/2), TATA-box binding protein-associated factor 15 (TAF15) and the viral protein ICP27, could interact with SRPKs and block their kinase activity (Fig. 2).

SAFB 1/2 contains an RRM domain and an RG/RGG motif, both of which mediates RNA recognition and binding [56, 57]. They can directly bind SRPK1 via C-terminal RG/RGG domain and repress its kinase activity [51]. The HSV-1 protein ICP27 also interacts with SRPK1 via N-terminal RGG box-containing domain, altering its localization and activity, leading to aberrant SR protein phosphorylation, and disrupting the spliceosome assembly [52, 58]. TAF15, a protein involved in transcription, splicing and RNA transport, binds SRPK1 through its C-terminal RGG domain, decreasing SRPK1 kinase activity. Overexpression of this RGG domain causes hypo-phosphorylation of SR proteins [59]. Other RG/RGG-containing proteins, including nucleolin, HNRPU and HNRNPA2B1, also inhibit SRPK1 activity, highlighting the key role of the RG/RGG motif in negatively regulating SRPK1 activity [59].

A recent study revealed that PIM-1 L, which is the long isoform of PIM-1, interacts with SRPK1 through its SR/SH-rich domain, leading to the inactivation of SRPK1 (Fig. 2) [60, 61]. Additionally, proteins that interact with the RS domain of SRPK1 substrates can mask phosphorylation sites and function as SRPK1 inhibitors. For example, the splicing factor-associated protein p32 binds unmodified SRSF1 and partially inhibits its phosphorylation by SRPK1 (Fig. 2) [62].

#### SRPKs activators

Enhancer of rudimentary homolog (ERH) directly interacts with the C-terminal region of SAFB1/2, abolishing their inhibitory effect on SRPK1 activity without affecting their role in transcription (Fig. 2) [63]. Silencing ERH enhancing the binding between SAFB and SRPK, leading to a significant reduction in SR protein phosphorylation. Conversely, ERH overexpression reduces the SRPK1-SAFB interaction, resulting in increased phosphorylation of SR proteins (Fig. 2) [63]. Activated AKT binds to and stimulates SRPK1 autophosphorylation, altering its interaction with molecular chaperones. This promotes SRPK1 nuclear translocation and enhances SR proteins phosphorylation (Fig. 2) [64].

### CLK family kinases

The CLK family of kinases, also known as the “LAMMER” family, comprises four homologues: CLK1, CLK2, CLK3, and CLK4 [65]. They are exclusively localized in the nucleus, where they phosphorylate the SR proteins (Fig. 2) [23]. CLKs share a common structural framework, featuring an N-terminal region characterized by the abundance of arginine/serine (RS) dipeptides, and a C-terminal kinase domain. While the C-terminal kinase domain exhibits high conservation across all family members, the N-terminal region varies among them [23, 66]. The CLKs RS-rich N-terminal domain is considered a bridge mediating the interaction between their C-terminal kinase domain and the RS domain of the SR proteins [23].

Emerging studies highlight CLKs as potential cancer therapeutic targets. CLK1 is overexpressed gastric cancer, pancreatic cancer, and high-grade gliomas, promoting cancer progression via modulating phosphorylation of spliceosome-related proteins [67–69]. CLK2 functions as an oncogenic kinase in breast cancer and colorectal cancer [70, 71]. Targeting the CLK2/SRSF9 axis regulates the splicing of androgen receptor splice variant-7 (ARV7) and decreases its expression in prostate cancer, potentially overcoming the resistance to androgen receptor signaling inhibitors [72]. CLK3 is upregulated in cholangiocarcinoma (CCA) and colorectal cancer [73, 74]. Its depletion suppresses colorectal tumor growth in vivo [74]. CLK4, highly expressed in invasive breast cancer, is linked to poor overall survival in mesenchymal-like triple-negative breast cancer. Its knockdown impairs TGF-β-driven epithelial-mesenchymal transition (EMT), suppressing cancer progression [75].

#### CLKs and SRPKs work on different layers of SR protein phosphorylation

Emerging studies have highlighted the key role of CLKs in regulating SR protein function. Unlike SRPK1, which phosphorylates only the Arg-Ser repeats in SRSF1, CLK1 targets both Arg-Ser and Ser-Pro dipeptides (Fig. 1b) [76]. The differential substrate specificity enables CLKs an alternative way to regulate SR proteins, which often contain multiple proline residues in the RS domain. These prolines are essential for high-affinity binding and phosphorylation of SRSF1 by CLK1 (Table 2) [76]. In addition, SRSF2 and SRSF5 are also shown to be phosphorylated by CLK1 at Ser-Pro dipeptides [77]. Notably, such modifications are beyond the capability of SPRKs, highlighting the unique role of the CLK family in the distinctive phosphorylation pattern of SR proteins. Furthermore, CLKs differ from SRPKs in their substrate recognition mechanisms. SRPK1 uses a classic docking groove in the C-terminal kinase domain to recognize and phosphorylate the RS domain of SRSF1 [78]. However, CLK1 lacks this docking groove and instead relies on its disordered non-catalytic N-terminal region, which binds to SRSF1 with high affinity [54, 79]. This N-terminal region promotes oligomerization, a process essential targeting SR proteins and directing CLK1 to nuclear speckles (Table 2) [79]. Removal of the N-terminus results in monomer formation and abolishes CLK1’s specificity for SR proteins.

**Table 2:** Comparison of CLKs and SRPKs in regulating SR protein phosphorylation

	SRPKs	CLKs
Family members	SRPK1, SRPK2, SRPK3	CLK1, CLK2, CLK3, CLK4
Localization	Nucleus and cytoplasm	Nucleus
Substrate specificity	Arg-Ser dipeptides	Both Arg-Ser and Ser-Pro dipeptides
Substrate recognition mechanism	Use a docking groove in the C-terminal kinase domain	Lack a docking groove and instead rely on disordered N-terminal to bind SR proteins with high affinity
Role in SR protein phosphorylation	In the cytoplasm, partially phosphorylate SR protein, promoting its nuclear import	In the nucleus, phosphorylate SR protein, facilitating its release from nuclear speckles
	Interaction between the N-terminus of CLK1 and nuclear SRPK1 further promotes SR protein phosphorylation, contributing to its diffusion from nuclear speckles and promoting spliceosome assembly	

Despite their distinct substrate specificities, SRPKs and CLKs act in a coordinated manner to regulate SR protein phosphorylation and splicing. SRSF1 is first partially phosphorylated by SRPKs in the cytoplasm, promoting its nuclear import mediated by TRN-SR (Fig. 2). Once in the nucleus, CLK1 phosphorylates Ser-Pro dipeptides near the C-terminus RS domain, facilitating the release of SRSF1 from nuclear speckles (Table 2) [76, 80, 81]. This increases the nucleoplasmic concentration of SR proteins, enhancing their availability for spliceosome assembly (Fig. 2) [82, 83]. Notably, interaction between the N-terminus of CLK1 and nuclear SRPK1 further promotes CLK1-mediated phosphorylation of Ser-Pro dipeptides in SRSF1, contributing to its diffusion from nuclear speckles and the subsequent promotion of spliceosome assembly (Fig. 2) [24, 83]. This hyper-phosphorylated form of SRSF1 is critical for recruiting the U1-snRNP to the 5’ splice site, a key step in spliceosome initiation [84].

### PRPF family kinases

Pre-mRNA processing factors (PRPFs belong to a family of serine/threonine protein kinases. The human homologue of PRPF4 (hPRPF4) colocalizes with SR proteins in nuclear speckles and interacts with CLK1. Additionally, the subnuclear localization of hPRP4 is dependent on the CLK1 kinase activity [85]. PRPF4B (also known as PRP4K) can phosphorylate the RS domain of SRSF1, and its depletion reduces SRSF1 phosphorylation, leading to decreased stability of the spliceosome B complex (Fig. 2) [86].

### DYRK family kinases

The DYRK family of kinases consists of five members: DYRK1A, DYRK1B, DYRK2, DYRK3 and DYRK4, with DYRK1A being the most extensively studied in RNA splicing and SR protein phosphorylation (Fig. 2) [87, 88]. DYRK1A is distributed throughout the nucleoplasm and accumulates in nuclear speckles where it colocalizes with SRSF2 [89]. Overexpression of DYRK1A induces disassembly of nuclear speckles, enhances SR protein phosphorylation, and causes redistribution of SR proteins [89]. Additionally, DYRK1A phosphorylates SRSF2 in vitro, and its overexpression suppresses SRSF2’s ability to promote tau exon 10 inclusion [90]. DYRK1A also interacts with SRSF1 and contributes to its phosphorylation at multiple serine residues (227, 234, and 238), facilitating its localization to nuclear speckles [91]. Moreover, DYRK1A interacts with and phosphorylates SRSF7 at multiple serine residues within the RS domain in rat brain extract and COS7 cells, leading to SRSF7 translocation from the nucleus to the cytoplasm [92]. Although DYRK1B shares 85% sequence homology with DYRK1A within their conserved kinase domain, little is known about the role of DYRK1B in SR protein phosphorylation [88].

### Cellular signaling pathway-related kinases

#### PI3K/AKT pathway

AKT, also known as protein kinase B (PKB), is a serine/threonine kinase that plays a key role in the PI3K/AKT signaling pathway. There are three isoforms, AKT1, AKT2 and AKT3, all of which are activated upon phosphorylation [93]. Once activated, AKT phosphorylates a wide range of downstream targets, influencing various cellular processes, including RNA splicing regulated by SR proteins, as discussed below.

Study has revealed that the RS domains of SRSF1 and SRSF7 can be phosphorylated in vitro by activated AKT, whereas not by its inactivated form, confirming that AKT functions as an SR protein-related kinase, with its activity being essential for this modification [94]. Another study showed that SRSF4 and SRSF2 (also known as SC35) are phosphorylated by AKT2 in response to insulin and serum exposure, which can be blocked by PI3K/AKT pathway inhibitors (Fig. 2) [26]. Similarly, SRSF5 (also known as SRp40) can be phosphorylated on Ser86 by AKT, upon PI3K pathway activation by insulin treatment [95]. Additionally, acinus, an SR-related protein essential for RNA splicing, is a direct AKT downstream target. Nuclear AKT-mediated phosphorylation of acinus alters of its subnuclear localization and biological function [96, 97].

Furthermore, AKT also indirectly regulate SR protein phosphorylation by modulating the activity of CLKs and SRPKs (Fig. 2). All CLK family members contain AKT-substrate motifs, suggesting they are regulated by the PI3K/AKT pathway. For example, in response to insulin, AKT2 enhances CLK1 activation and promotes CLK1-mediated phosphorylation of SRSF4 and SRSF6 [26]. Mutation of these AKT phosphorylation motifs in CLK1 reduces SR protein phosphorylation, further supporting the AKT’s regulatory role [98]. Additionally, activated Akt binds to and stimulates SRPK1 autophosphorylation, altering in its interaction with molecular chaperones and enhancing SRPK1 nuclear translocation and SR protein phosphorylation (Fig. 2) [64]. Another study reported that AKT inhibition decreases SRPK1 and SRPK2 expression and reduces SRSF6 phosphorylation. Combined AKT and SRPK inhibition exhibits a synergistic effect, significantly decreasing the phosphorylation of SRSF4, SRSF5, and SRSF6 [99]. Intriguingly, SRPK1 overexpression can, in turn, increase AKT phosphorylation, contributing to the activation of the downstream NF-κB pathway, whereas SRPK1 depletion reduces AKT phosphorylation, which may explain the synergy of AKT and SRPK inhibition (Fig. 2) [100].

#### MAPK pathway

The family of the mitogen-activated protein kinases (MAPKs) comprises seven distinct members, with JNKs, ERKs and p38 isoforms being the most widely studied [101]. While MAPKs are known to regulate various splicing factors, their direct role in modulating SR proteins remains limited [102, 103]. An early study suggested an indirect regulatory mechanism, showing that MKK_3/6_-p38 signaling modulates hnRNP A1, a functional antagonist of SR proteins. This alters the nuclear hnRNP A1/SRSF1 ratio, impacting alternative splicing (Fig. 2) [104].

More recently, SRSF1 was found to influence the MNK/p38 MAPK axis and, thereby modulating cellular homeostasis and cytokine function [105, 106]. Additionally, MAPK-interacting kinase (MNK) activity has been shown to modulate SRPK activity indirectly through negatively regulating mTORC2 and AKT, providing further support for AKT’s role in SRPK activity regulation as mentioned above (Fig. 2) [107].

#### cAMP signaling pathway

Protein kinase A (PKA), also known as cAMP-dependent protein kinase, is activated by intracellular cAMP levels and regulated through interaction with A-kinase anchoring proteins (AKAPs) [108]. AKAP17A has been identified as a novel AKAP capable binding PKA both in vitro and in vivo [109]. It colocalizes with the PKA catalytic subunit and SRSF2 in nuclear speckles. Additionally, PKA interacts with SRSF1, modulating its phosphorylation and subsequent binding activity to tau pre-mRNA (Fig. 2) [110].

### Additional kinases regulating SR protein phosphorylation

#### DNA topoisomerase I

DNA topoisomerase I (Topo I), an enzyme that relaxes DNA supercoiling during transcription, has also been identified as a potential SR proteins kinase [111, 112]. Although it lacks a canonical ATP-binding domain, Topo I can phosphorylate specific serine residues within the RS domains of SR and SR-related proteins, through an ATP-dependent mechanism (Fig. 2). However, the reaction and kinetics of Topo I-mediated phosphorylation differ from those of SRPK1 [113].

Topo I depletion results in hypo-phosphorylation of SR proteins and impairs exonic splicing enhancer (ESE)-dependent splicing, highlighting its essential role in the ESE recognition by SR proteins [114]. Notably, Topo I colocalizes with SRSF6 and modulates its phosphorylation (Fig. 2) [115]. In turn, phosphorylated SRSF6 drives Topo I recruitment to the active transcription site, facilitating efficient mRNA release.

#### Aurora kinase A

Aurora kinase A (AURKA), a member of the serine/threonine kinases family, plays key role in chromosome segregation and cytokinesis [116]. Previous studies have shown that pharmacological inhibition or knockdown of AURKA resulted in the downregulation of SRSF1 protein level, without affecting other SR proteins or splicing factors [117]. A recent study also reported AURKA colocalization with SRSF2 in nuclear speckles in U2OS cells (Fig. 2). Moreover, AURKA interactions with SRSF1, SRSF3 and SRSF7 were identified via proteomics and co-immunoprecipitation. In vitro kinase assays further confirmed that AURKA directly phosphorylates these SR proteins [116].

#### snRNP-associated kinase

An early study has demonstrated the enzymatic activity of the snRNA-associated kinase, which is responsible for the phosphorylation of U1-70K within its C-terminal RS domain [118]. Similarly, this snRNP-associated kinase also phosphorylates the RS domain of SRSF1 in in vitro assays (Fig. 2) [118]. Another study reported that U5-100kD, which contains an N-terminal RS domain, can be phosphorylated in vitro by both CLK1 and the snRNP-associated kinase [119].

## Regulation of SR protein dephosphorylation by phosphatases

As introduced earlier, besides of phosphorylation, SR proteins also require dephosphorylation to ensure the splicing processing. Dephosphorylation of SR proteins, where phosphates are removed from the RS domains, is primarily mediated by serine/threonine protein phosphatases [120]. In eukaryotes, protein phosphatase 1 (PP1) and protein phosphatase 2A (PP2A), which belong to the phosphoprotein phosphatase (PPP) superfamily of serine-threonine phosphatases, contribute to more than 90% of the protein phosphatase activity [121]. At present, it is suggested that PP1 forms stable distinct complexes with approximately 650 proteins, while only around 70 PP2A complexes are formed, indicating that PP1 participates in most of the protein dephosphorylation activities in the cell [121].

### PP1

PP1 is an oligomeric enzyme that consists of a catalytic subunit (PP1c) but lacks inherent substrate specificity. Through interacting with various regulatory proteins, PP1 achieves specificity and forms highly specific holoenzymes [122]. The PP1c subunit is encoded by three distinct genes: PPP1CA, PPP1CB and PPP1CC. In the context of cancer, amplification of these three PP1c-encoding genes is one of the most observed alterations [123]. For example, amplification of PP1CA is notably enriched in metastatic prostate cancer, indicating its potential role as a pro-metastatic gene in this malignancy [124]. Conversely, downregulation of PPP1CA and PPP1CC has been reported to correlate with poorer prognosis in breast cancer [122]. Since PP1c forms distinct holoenzymes with various regulatory proteins across different cellular contexts, its role is largely dependent on its interactors [123]. For example, the interaction between PPP1CA and tensin 1 inhibits cancer cell migration and invasion, whereas the interaction of PP1 with TGF-β promotes malignant transformation [125]. These findings suggest the complex and context-dependent functions of PP1 in cancer development and progression.

As PP1 plays a crucial role in protein dephosphorylation in mammalian cells, it is the most extensively studied protein phosphatase in regulating SR protein dephosphorylation (Fig. 2) (Table 3). PP1 significantly reduces SR protein phosphorylation in Hela cells and facilitates the subnuclear redistribution of splicing factors. It interacts weakly with SRSF1 and dephosphorylates it inefficiently, whereas SRPK1 forms a high-affinity complex with SRSF1 and rapidly phosphorylates its RS1 domain [28]. The RRM domain of SRSF1 protects against PP1-mediated dephosphorylation, and its binding to PP1 can repress phosphatase activity, thereby promoting RS domain phosphorylation [126]. In addition to SRSF1, PP1 also binds to and dephosphorylates SRSF10 (also known as SRp38) upon dissociation from its inhibitor, nuclear inhibitor of PP1 (NIPP), during heat shock. However, under normal conditions, PP1 is restrained by NIPP (Fig. 2). Furthermore, the interaction between SRSF10 and 14–3-3 proteins also shields SRSF10 from dephosphorylation [127].

**Table 3:** Phosphatases regulating SR protein dephosphorylation and functions

Phosphatases	Effects on SR protein function	Dephosphorylation features	Direct/indirect regulation	Well-studied SR protein targets
Protein phosphatase 1 (PP1)	Major phosphatase mediating SR protein dephosphorylation; promotes SR protein redistribution; alters spliceosome dynamics; switches SR proteins to splicing-repressive states	Activity of RS-domain dephosphorylation controlled by regulatory proteins (e.g., NIPP, 14–3-3)	Direct	SRSF1, SRSF10
Protein phosphatase 2A (PP2A)	Contributes to SR protein dephosphorylation in specific contexts; induces SR protein hypo-phosphorylation and functional inactivation; links splicing with translational control	Context-dependent; RS-domain dephosphorylation via specific holoenzymes	Direct	SRSF1

Given PP1 has been shown to function as a ceramide-activated protein phosphatase, exogenous ceramide treatment induces the dephosphorylation of SR proteins. Endogenous ceramide generated upon activation of the Fas cell-surface death receptor (also known as APO-1 or CD95), a central mediator of apoptosis, activates PP1 and promotes dephosphorylation of SR proteins (Fig. 2) [128]. In contrast to natural ceramides, the synthetic analog C6 pyridinium ceramide (PyrCer) binds to and inhibits PP1, thereby blocking the dephosphorylation of splicing factors, including SRSF1 (Fig. 2) [129].

### PP2A

In addition to PP1, PP2A also play a role in dephosphorylation of SR proteins. PP2A comprises three functional subunits: a catalytic C subunit (PP2Ac) and a scaffold A subunit, which together form the core of the enzyme [130]. Like PP1, PP2A assembles into distinct holoenzyme complexes by interacting with more than 40 substrate-specifying B regulatory subunits [130]. These B regulatory subunits exhibit tissue-specific expression, mediating diverse substrate specificities and participating in various cellular signaling cascades [131]. PP2A is generally considered as a tumor suppressor because it inactivates multiple oncogenic drivers such as MYC [132]. However, depending on the specific composition of its holoenzyme, PP2A can also exhibit pro-tumorigenic functions [132, 133]. Despite this context-dependent behavior, pharmacological inhibition of PP2A has demonstrated promising anti-cancer effects through impairing DNA repair mechanisms, particularly when combined with radiotherapy or other therapeutic agents such as DNA damaging drugs [134].

In contrast to PP1, which interacts with numerous proteins and has been extensively studied, research on the role of PP2A in SR protein dephosphorylation remains limited (Table 3) [121]. An early study reported that the virus-encoded protein E4–ORF4 activates the PP2A-dependent dephosphorylation of SR proteins in HeLa cells, leading to their hypo-phosphorylation and inactivation (Fig. 2) [135]. SRSF1 can act as an adaptor, interacting with both PP2A and mTOR, promoting the hyperphosphorylation of 4E-BP, thereby regulating translation initiation [136].

Increasing evidence indicates that activities of PP1 and PP2A are closely coordinated through cross-regulatory mechanisms. They can colocalize to the same substrates or molecular compartments, such as kinetochores or cell cycle regulators, while exhibit distinct and sometimes opposing functions through differential recruitment by regulatory subunits [137, 138]. Phosphorylation signals differentially regulate PP1 and PP2A recruitment, inhibiting PP1 while facilitating PP2A association, thereby establishing feedback loops that enable precise temporal and context-specific regulation [137]. Such cross-regulatory mechanisms may contribute to the control of SR protein dephosphorylation, with PP1 and PP2A potentially acting in a coordinated and context-dependent manner to modulate splicing factor activity and RNA processing.

## Therapeutic potential of targeting SR protein phosphorylation

To date, small-molecule inhibitors targeting SRPKs and CLKs are the most extensively developed and evaluated for cancer therapy. Here, we summarize their anti-cancer efficacy and roles in regulating in SR protein phosphorylation and RNA splicing. Inhibitors that have not yet been tested in cancer cells or have not been implicated in the regulation of SR proteins are not included in this review.

### Small molecule SRPK inhibitors regulating SR protein phosphorylation in cancer

Several small-molecule inhibitors targeting SRPKs have been developed to regulate SR protein phosphorylation and aberrant alternative splicing in cancer, thereby suppressing tumor cell growth, angiogenesis, and survival.

**SPHINX**, a selective SRPK1 inhibitor, significantly reduced cell viability in acute myeloid leukemia cell lines and enhances the efficacy of chemotherapeutic agents such as azacitidine [139]. It inhibits SR protein phosphorylation, shifting vascular endothelial growth factor (VEGF) splicing toward the anti-angiogenic isoform VEGF165b and suppressing tumor growth in prostate cancer in vivo [140].

Similarly, **SRPKIN-1**, a SRPK1/2 inhibitor designed based on an anaplastic lymphoma kinase (ALK) inhibitor, attenuates SR protein phosphorylation, and promotes VEGF165b splicing, demonstrating its anti-cancer potential [141].

**SPHINX31,** a more potent SRPK1 inhibitor, and **SRPIN340**, a dual SRPK1/2 inhibitor, suppress SR protein phosphorylation and induce apoptosis in cholangiocarcinoma (CCA) cells by downregulating anti-apoptotic BIN1+12A and upregulating pro-apoptotic MCL-1S and BCL-xS [49]. Additionally, SPHINX31 was shown to downregulate the expression of pro-angiogenic isoform of VEGF in CCA cells [142]. A Phase I/II clinical trial demonstrated the selectivity of EXN407 (an eye-drop formulation of SPHINX31) for targeting the pro-angiogenic isoform of VEGF that drives aberrant growth of leaky blood vessels in the eyes of patients with diabetic macular oedema. EXN407 was characterized as safe and well tolerated, with no major or serious adverse events reported that were attributable to the treatment (NCT04565756). However, to date, no clinical studies have evaluated the efficacy of this compound in cancer therapy.

**SCO-101** (also known as Endovion), a recently characterized SRPK1 inhibitor which also inhibits drug efflux pump ABCG2, reduces SR protein phosphorylation and causes nascent transcriptional changes like those induced by SRPIN340 [143]. A Phase I clinical trial (NCT04652206) reported that the combination of SCO-101 with gemcitabine and nab-paclitaxel was well tolerated in patients with unresectable or metastatic PDAC. However, no clear added efficacy was observed with these combinations [144, 145]. In addition, a Phase II clinical trial (NCT04247256) is being conducted to evaluate the safety, tolerability, and anti-tumor effect for SCO-101 in combination with FOLFIRI in metastatic colorectal cancer (mCRC) patients with acquired resistance to FOLFIRI.

Furthermore, targeting the SRPK docking groove to block the interaction between SRPK and SR proteins offers an alternative strategy to inhibit SRPK catalytic activity. **DBS1** disrupts SRPK–SRSF1 interaction, reducing SRSF1 phosphorylation and shifting VEGF splicing to VEGF165_b_ [146]. **C-DBS,** a covalent PPI inhibitor of SRPKs developed from DBS1, exhibits a significantly lower IC_50_ and effectively suppresses SR protein phosphorylation, angiogenesis, cell migration and invasion in vitro [147].

Table 4 summarizes the SRPK inhibitors discussed in this review.

**Table 4:** Summary of the small-molecule SRPK inhibitors discussed in the current review and their key effects

Inhibitor	Target(s)	IC_50_(nM)	Key effects	Clinical status
SPHINX	SRPK1	≈580	↓ SR protein phosphorylation; ↓ cell viability in AML cells [139] Shifts VEGF splicing to VEGF165b; ↓ tumor growth in prostate cancer [140]	Preclinical status
SRPKIN-1	SRPK1/2	SRPK1: 35.6 SRPK2: 98	↓ SR protein phosphorylation; shifts VEGF splicing to VEGF165b [141]	Preclinical status
SPHINX31	SRPK1	≈5.9	↓ SR protein phosphorylation; ↓ BIN1+12A; ↑MCL-1S and BCL-xS; ↑ apoptosis in cholangiocarcinoma (CCA) cells [49] ↓ pro-angiogenic VEGF165a isoform in CCA cells [142]	Phase I/II: to evaluate the safety and tolerability of EXN407 (an eye-drop formulation of SPHINX31) in subjects with diabetic macular oedema (NCT04565756, completed).
SRPIN340	SRPK1/2	890	↓ SR protein phosphorylation; ↓ BIN1+12A; ↑MCL-1S and BCL-xS; ↑ apoptosis in CCA cells [49]	Preclinical status
SCO-101	SRPK1/ ABCG2	Not specified	↓ SR protein phosphorylation; ↑ nascent transcriptional changes [143]	Phase I/II: to investigate safety, tolerability, and maximum tolerated dose for SCO-101 in combination with gemcitabine and nab-paclitaxel in inoperable pancreatic cancer patients (NCT04652206, unknown status) Phase II: to evaluate the safety, tolerability, and anti-tumor effect for SCO-101 in combination with FOLFIRI in metastatic colorectal cancer (mCRC) patients with acquired resistance to FOLFIRI (NCT04247256, unknown status)
DBS1	SRPK1/2	SRPK1: 3.2 ±1.5 µM (Kd) SRPK2: Not specified	Disrupts SRPK-SRSF1 interaction; ↓ SR protein phosphorylation; shifts VEGF splicing to VEGF165b [146]	Preclinical status
C-DBS	SRPK1/2	SRPK1: 402 ± 19 (Kd) SRPK2: Not specified	↓ SR protein phosphorylation; ↓ angiogenesis, cell migration and invasion [147]	Preclinical status

### Small molecule CLK inhibitors regulating SR protein phosphorylation in cancer

Several small-molecule inhibitors targeting CLKs have been developed to regulate SR protein phosphorylation and aberrant alternative splicing in cancer, demonstrating significant antitumor activity across multiple cancer types.

**TG003**, a benzothiazole compound, inhibits SR protein phosphorylation and reduces prostate cancer cell proliferation, migration, and invasion [148]. A recent study has demonstrated that TG003 could reverse platinum resistance of ovarian cancer (OC) cells. TG003 in combination with PARP inhibitor synergistically suppresses tumor growth in OC BRCA1^WT^ patient-derived xenograft model [149].

**T-025** is a potent, orally available CLK inhibitor that suppresses SR protein phosphorylation and modulates splicing, hindering tumor cell proliferation, particularly effective in MYC-driven cancers [150, 151]. It also restricts dynamic mobility of SR protein and disrupts SR protein’s function during spliceosome assembly, blocking cell growth and migration in triple-negative breast cancer (TNBC) [151].

**Cpd-2** and **Cpd-3** were identified through high-content screening and exhibited the strongest inhibitory activity against CLK2. Both compounds reduce SRSF1 and SRSF4 phosphorylation in a dose-dependent manner, resulting in widespread alterations in alternative splicing, inhibition of breast cancer cell growth, and promotion of apoptosis [152].

**T3** exhibits significant inhibitory activity against CLK1, CLK2 and CLK3. It decreases the phosphorylation of SRSF1, SRSF4 and SRSF6, leading to defects of exon recognition and increased exon skipping [153]. Additionally, it induces apoptosis in ovarian cancer cells and causes cell cycle arrest in colorectal cancer cells. When combined with Bcl-xL/Bcl-2 inhibitors, T3 synergistically enhances apoptosis in cancer cells [154].

**Compound 25** shows strong inhibitory activity against CLK1, CLK2 and CLK4. It induces the redistribution of SR proteins from nucleoplasm to nuclear speckles and promotes autophagy in OC cell lines in a dose-dependent and time-dependent manner [155].

**1C8** and **GPS167** inhibit cell proliferation in a panel of cancer cell lines, affects CLK activity and SRSF10 phosphorylation, microtubule function and EMT, implicating their potential to combat tumor progression and metastasis [156, 157].

**KuWal151**, an inhibitor of CLK1, CLK2 and CLK4, shows broad anti-proliferative effects across cancer cell lines. However, it also affects cell proliferation in non-cancerous cells and its influence on SR protein phosphorylation and splicing remains under-investigated [158].

**CC-671**, a dual TTK (also known as Mps1, monopolar spindle 1 kinase)/CLK2 inhibitor, blocks the SRSF4 phosphorylation and alters alternative splicing, significantly inhibiting tumor growth and inducing apoptosis in TNBC [159]. While interestingly, a study revealed a strong antagonistic interaction between CC-671 and paclitaxel in patient-derived multi-cell type tumor spheroids [160].

**SM08502** (also known as Cirtuvivint), a pan-CLK inhibitor, suppresses gastrointestinal tumor growth by reducing SR protein phosphorylation and downregulating Wnt pathway-related genes [161]. Compared to CC-671, it shows stronger cytotoxicity in patient-derived tumor spheroids [160]. Two Phase I clinical trial studies, which aimed to evaluate the safety and pharmacokinetics of orally administered SM08502 (NCT03355066), or in combination with hormonal therapy or chemotherapy (NCT05084859), in subjects with advanced solid tumors, have been terminated due to business reasons by sponsor. Orally administered SM08502 demonstrated proof of mechanisms in subjects with advanced solid tumors by inhibiting CLK1 and disrupting alternative splicing at tolerated doses. However, adverse effects such as nausea, diarrhea, fatigue, and vomiting were observed [162]. A Phase I clinical trial testing SM08502 and its combination with ASTX727 to improve the outcomes of patients with acute myeloid leukemia (AML) and myelodysplastic syndromes (MDS) is recruiting (NCT06484062). Additionally, a Phase II clinical trial of SM08502 as a second-line treatment for advanced soft tissue sarcomas is being conducted (NCT07032285).

**CTX-712** (also known as Rogocekib), a pan-CLK inhibitor, shows strong inhibition on SR protein phosphorylation, cell proliferation and induces global splicing changes in lung cancer and human myeloid cell lines. Its in-vivo anti-tumor efficacy was also demonstrated [163, 164]. A Phase I/II clinical study evaluating CTX-712 in patients with relapsed/refractory AML and higher risk MDS is currently ongoing (NCT05732103). It has demonstrated good tolerability and preliminary anti-cancer efficacy in patients with hematologic malignancies [165].

**SGC-CLK-1 (CAF-170)**, a potent inhibitor of CLK1, CLK2, and CLK4, exhibits binding affinity to CLK1, 2 and inhibits SR protein phosphorylation, altering its subcellular distribution and reducing cell viability in multiple cancer types [166].

**LBM22**, a recently discovered CLK inhibitor, induces apoptosis and inhibits non-small cell lung cancer (NSCLC) cell viability by decreasing SR protein phosphorylation [167].

**Compound 670551**, identified via structure-based virtual screening, selectively targets CLK2, suppressing TNBC cell growth and promoting apoptosis through downregulating SR protein phosphorylation [168].

A summary of the CLK inhibitors discussed above is provided in Table 5.

**Table 5:** Summary of the small-molecule CLK inhibitors discussed in the current review and their key effects

Inhibitor	Target(s)	IC_50_(nM)	Key effects	Current status
TG003	CLK1/2/4	CLK1: 20 CLK2: 200 CLK4: 15	↓ SR protein phosphorylation; ↓ cell proliferation, migration, and invasion in prostate cancer (PCa) [148] Reverses platinum resistance; ↓ tumor growth when in combination with PARP inhibitor in BRCA1^WT^ ovarian cancer (OC) [149]	Preclinical status
*T*-025	CLK1/2/3/4, DYRK1A/B, DYRK2	CLK1: 4.8 (Kd) CLK2: 0.096 (Kd) CLK3: 6.5 (Kd) CLK4: 0.61 (Kd) DYRK1A: 0.074 (Kd) DYRK1B: 1.5 (Kd) DYRK2: 32 (Kd)	↓ SR protein phosphorylation; ↓ cell proliferation, mainly in MYC-driven cancers; ↓ tumor growth in breast cancer [150] ↓ SR protein dynamic mobility; ↓ cell growth and migration in triple-negative breast cancer (TNBC) [151]	Preclinical status
Cpd-2	CLK1/2	CLK1: 1.1 CLK2: 2.4	↓ SR protein phosphorylation; ↓ cell growth and ↑ apoptosis in breast cancer [152]	Preclinical status
Cpd-3	CLK1/2	CLK1: 1.1 CLK2: 2.1	↓ SR protein phosphorylation; ↓ cell growth and ↑ apoptosis in breast cancer [152]	Preclinical status
T3	CLK1/2/3	CLK1: 0.67 CLK2: 15 CLK3: 110	↓ SR protein phosphorylation; ↑ apoptosis in OC cells; ↑ cell cycle arrest in colorectal cancer cells; synergistic pro-apoptotic effects when combined with Bcl-xL/Bcl-2 inhibitors [153–154].	Preclinical status
Compound 25	CLK1/2/4	CLK1: 2 CLK2: 31 CLK4: 8	↑ redistribution of SR proteins from nucleoplasm to nuclear speckles; ↑ autophagy in OC cells [155].	Preclinical status
IC8	CLK1/2/4, DYRK1A/B	Not specified	↓ SR protein phosphorylation; ↓ cell proliferation in a panel of cancer cell lines; impacts microtubule function and EMT [156]	Preclinical status
GPS167	CLK1/2/4	CLK1: 90 CLK2: 82 CLK4: 123	↓ SR protein phosphorylation; ↓ cell proliferation in a panel of cancer cell lines; impacts microtubule function and EMT [156, 157]	Preclinical status
KuWal151	CLK1/2/4	Not specified	↓ Cell proliferation cross cancer cell lines; also affects non-cancerous cells [158]	Preclinical status
CC-671	TTK/CLK2	CLK2: 6 TTK: 5	↓ SR protein phosphorylation; ↑ apoptosis; ↓ tumor growth in TNBC [159] Strong antagonistic interaction with paclitaxel in multi-cell type tumor spheroids [160]	Preclinical status
SM08502 (Cirtuvivint)	CLK1/2/3/4, DYRK1A/B, DYRK2, DYRK4	CLK1: 8 CLK2: 2 CLK3: 22 CLK4: 1 DYRKs: 2–13	↓ SR protein phosphorylation; ↓ Wnt pathway-related genes; ↓ gastrointestinal tumor growth [161] Cytotoxicity in patient-derived multi-cell type tumor spheroids [160]	Phase I: to evaluate its safety and pharmacokinetics, or in combination with hormonal therapy/chemotherapy, in advanced solid tumors (NCT03355066 and NCT05084859, both are terminated) Phase I: to test SM08502, and in combination with ASTX727 in treating patients with acute myeloid leukemia (AML) and myelodysplastic syndromes (MDS) (NCT06484062, recruiting). Phase II: evaluate SM08502 as a second-line treatment for advanced soft tissue sarcomas (NCT07032285, recruiting)
CTX-712 (Rogocekib)	CLK1/2/3/4, DYRK1A/B	CLK1: 0.69 CLK2: 0.46 CLK3: 3.4 CLK4: 8.1 DYRK1A: 1.1 DYRK1B: 1.3	↓ SR protein phosphorylation; ↓ cell proliferation in human myeloid cell lines; ↓ tumor growth in leukemia [164] ↓ SR protein phosphorylation; ↓ lung cancer cell proliferation; ↓ tumor growth in lung cancer [163]	Phase I/II: in treating patients with relapsed/refractory AML and higher risk MDS (NCT05732103, recruiting).
SGC-CLK-1 (CAF-170)	CLK1/2/4	CLK1: 13 CLK2: 4 CLK4: 46	↓ SR protein phosphorylation; ↓ cell viability in multiple cancer types [166]	Preclinical status
LBM22	CLK1/2/3/4	CLK1: 4.6 CLK2: 3.9 CLK3: 28.7 CLK4: 7.3	↓ SR protein phosphorylation; ↑ apoptosis; ↓ non-small cell lung cancer (NSCLC) cell viability [167]	Preclinical status
Compound 670551	CLK2	619.7	↓ SR protein phosphorylation; ↑ apoptosis; ↓ TNBC tumor growth and survival [168]	Preclinical status

## Future perspectives and conclusions

This review aims to provide a comprehensive overview of the current understanding of the regulatory mechanism governing SR protein phosphorylation. We outlined the complex network of kinases and phosphatases that modulate the dynamic balance between phosphorylation and dephosphorylation of SR proteins, highlighting the critical role of this balance in regulating RNA splicing programs.

Additionally, we summarized small-molecule SRPK and CLK inhibitors currently under-investigation for cancer treatment, illustrating their potential to combat tumor progression and metastasis through modulating SR protein phosphorylation and splicing. Worthy to note is that some potent SRPK/CLK inhibitors are not discussed in detail in this review despite demonstrating strong inhibition of kinase activity and SR protein phosphorylation. For example, MSC-1186, a pan-SRPK inhibitor, shows significantly higher selectivity and nanomolar cellular potency compared to SRPIN340, SPHINX31 and SRPKIN-1 [169]. However, its poor solubility renders it unsuitable for in-vivo studies. Further research should therefore prioritize the development of these highly selective inhibitors to minimize off-target toxicity, alongside chemical optimization strategies to improve pharmacokinetic properties such as solubility.

Given the essential role of SR protein phosphorylation in cancer and the complex regulatory network controlling this process, combination therapies targeting related kinases may offer promising new avenues for cancer treatment. Notably, dual inhibition of SRPK and CLK using SRPK inhibitor MSC-1186 in combination with CLK inhibitors CLK-T3 or *T*-025 results in more robust suppression of SR protein phosphorylation compared to treatment with either agent alone, whereas the exploration of anti-cancer potency is lacking (Table 6) [169]. In addition, a synergistic effect using SRPK or CLK inhibitors with chemotherapeutic agents has also been demonstrated (Table 6) [144, 154, 170]. For example, the combination of the SRPK1 inhibitor SPHINX31 or the dual SRPK1/2 inhibitor SRPIN340 with paclitaxel caused synergistic cytotoxicity in human paclitaxel-resistant pancreatic ductal adenocarcinoma (PDAC) cells. SCO-101 also exhibits similar synergistic effect with paclitaxel, potentially through inhibition of SRPK and/or drug efflux transporter ABCG2 (Table 6) [144]. Altogether, these studies provide evidence supporting the potential of using splicing modulators in combination with other chemotherapeutic agents for cancer treatment and encourage further exploration of novel and more effective drug combinations in the future.

**Table 6:** Examples of drug combinations discussed in the current review

Drug combinations	Key effects
• SRPK: MSC-1186 • CLK: CLK-T3 or *T*-025	More robust suppression of SR protein phosphorylation; anti-cancer potency is unknown [169]
• SRPK: SPHINX31 or SRPIN340 • Paclitaxel	Synergistic cytotoxicity in paclitaxel resistant PDAC cells [144]
• SRPK1/ABCG2: SCO-101 • Paclitaxel	Synergistic cytotoxicity in paclitaxel resistant PDAC cells, through inhibition of SRPK and/or drug efflux transporter ABCG2 [144]
• CLK/DYRK: SM08502 • Paclitaxel	Synergistic effects on cell proliferation inhibition and tumor growth reduction in endometrial cancer cell lines [170]
• CLK: T3 • Bcl-xL/Bcl-2: ABT-263	Synergistic effects on apoptosis induction in human colorectal cancer and ovarian cancer cells [154]

The clinical translation of splicing modulators faces additional challenges beyond specificity and solubility, as splicing is essential for normal cellular function and on-target toxicities remain a significant concern, particularly in highly proliferative cell types such as activated immune cells and hematopoietic stem cells [151]. Future studies should focus on strategies such as combination therapies and advanced drug delivery systems to selectively target tumor cells, enhance the therapeutic window, and improve the safety of splicing-targeted treatments, including SRPK and CLK inhibitors. Moreover, given their critical roles in splicing, continued research into SR proteins and their complex regulatory mechanisms in cancer will provide valuable insights to guide the development of novel and more effective therapies.

## Data Availability

Data availability is not applicable to this article as no new data were created or analyzed in this study.
